# Supplementary material for: Are carbon emissions trading and green financial instruments synergistic? -Comprehensive quantitative research based on content analysis
Source: PLoS One. 2024 Mar 7;19(3):e0298601. doi: 10.1371/journal.pone.0298601 (PMC10919601; doi:10.1371/journal.pone.0298601)
Supplement: S1 Data — (ZIP) [file pone.0298601.s001.zip › Data/地级市Z_F工作报告.zip!/╜¡╦╒/╠⌐╓▌╩╨/└┤╫╘╣1⁄2╓┌║┼í╛┬φ┐╦╩2╛▌═°í┐_2013.docx]

各位代表：

现在，我代表市人民政府向大会作工作报告，请予审议，并请市政协委员和其他列席人员提出意见。

一、2012年工作回顾

过去的一年，我们在中共泰州市委的正确领导下，坚持以邓小平理论、“三个代表”重要思想、科学发展观为指导，狠抓转型升级这一事关未来发展的根本，认真实施“八项工程”，深入推进“双轮驱动”，加快建设“三个名城”，统筹抓好稳增长、调结构、惠民生、促和谐等各项工作，全市经济社会发展呈现稳中有进的良好态势。

这一年，是我市历史上极不平凡的一年，大喜事不断。国家主席胡锦涛回家乡考察，省委、省政府正式确定我市为全省转型升级综合改革试点，泰州大桥正式通车，扬泰机场顺利通航，姜堰撤市设区获国务院批准，国家现代农业示范区成功获批，靖江经济开发区升格为国家级开发区，总投资102亿元的中海油气项目开工建设，总投资85亿元的国电泰州电厂二期获批建设。

经济平稳较快发展。积极应对复杂形势，把稳增长放在更加重要的位置，经济发展经受住严峻考验，在困境中实现了赶超。预计全年实现地区生产总值2700亿元，增长12.3%；三次产业增加值分别为190亿元、1435亿元和1075亿元，分别增长4.5%、13%和12.5%。公共财政预算收入233.25亿元，同口径增长15%。完成固定资产投资1455亿元，增长21.5%，其中工业投资增长25%。社会消费品零售总额742亿元，增长15%。外贸进出口总额102亿美元，实际利用外资13亿美元，外经营业额7.2亿美元，增长6%。农业现代化建设加快推进。新增高效设施农业20万亩、高效设施渔业4万亩、高标准农田11.8万亩，新建三大合作组织850家，农业利用“三资”40亿元，农业综合机械化水平达75%，粮食生产实现“九连增”，粮食单产、淡水产品产量保持全省第一。兴化现代农业产业园区、靖江生祠现代农业示范园区被认定为省级园区，姜堰溱湖河蟹产业园区获批省级农业标准化示范区，靖江、海陵、高港通过省级基本实现水稻种植机械化县（市）验收。工业经济运行质态良好。规模以上工业实现销售6800亿元、利税780亿元、利润480亿元，分别增长18%、16%和16%，工业经济综合效益指数位居全省前列。民营经济更趋活跃，新增私营企业7000家、个体工商户25000户，新增私营个体经济注册资本300亿元。建筑业实现总产值1800亿元，工程结算收入首次过千亿元。现代服务业持续发展。8大服务业集聚区、30强企业分别实现营业收入601亿元和215亿元，30个重点项目完成投资105亿元，服务业地税收入占比达62%，新增3家省服务业百强企业、4家省服务业创新百强企业，总投资86亿元的天星洲港口物流园等项目签约落户，姜堰镇村服务标准化项目入围国家级服务业标准化试点。金融业加快发展，年末全市本外币存款余额3000亿元，贷款余额2080亿元，分别增长13.9%和14.9%，新增直接债务融资100亿元，在全省率先实现村镇银行和异地支行县域全覆盖。旅游品牌影响进一步扩大，我市荣获“长三角最佳慢生活旅游名城特别奖”，溱湖风景区获批国家5A级旅游景区，全市接待游客1550万人次，实现旅游总收入175亿元。

转型发展步伐加快。新兴产业贡献份额逐步提高。着力构建“1+3+N”产业体系，全市生物技术与新医药、新能源、电子信息三大新兴产业实现利税150亿元、利润90亿元，分别增长30%和38%，占规模以上工业的18.8%和19.2%，提高3个和2.1个百分点。坚持用高新技术和先进适用技术改造提升传统产业，实现高新技术产业产值2500亿元，增长23%，占规模以上工业的35.2%。产业园区的集聚效应加快放大。围绕打造高品质特色产业园区，推进“一城一区一园一带一块”产业发展和功能布局。继续举全市之力建设中国医药城，建成国家新型工业化产业示范基地，新落户企业180家，落地申报医药创新成果103个，阿斯利康、武田制药等产业项目进展加快，成功举办第六届中国生物产业大会、第三届国际医药博览会。出口加工区二期获批，纬创资通追加投资1.8亿美元，导光板、触摸屏等产品和项目顺利投产。新能源产业园金钢切割线项目投产，新引进汉能TCO导电玻璃、AA集团风光电互补等一批重大项目，成功举办全国新能源产业高层研讨会。沿江产业带建设纵深推进，新签约过亿美元项目12个，核心港区永安港务三期集装箱码头主体竣工，靖江港获批国家一类独立对外开放口岸。里下河特色经济板块加快发展，姜堰众品食业等农副产品深加工项目竣工投产，可可、安井、伽力森等一批知名食品企业落户兴化。泰州新技术船舶基地、新能源产业园获批国家级特色产业基地，高港高新区获批中国通用电力产业基地，靖江核电装备、泰兴现代装备科技产业园获批省级科技产业园。企业发展的影响力不断扩大。出台“工业大企业（集团）培育计划”，新增销售10亿元以上企业20家、利税过亿元企业14家、上市企业2家。加强企业技术改造，实施亿元以上重点项目275个，9个项目列入国家产业振兴和技术改造投资计划。引导企业加大研发投入，加快研发机构建设，新公示国家高新技术企业73家，7家企业入选省首批创新型领军企业培育库。支持企业加强新品开发和品牌建设，新增国家重点新产品9项、省级高新技术产品326项、省名牌产品20个、中国驰名商标2件，77家企业参加191个项目的国家和行业标准制修订，70家企业获批国家标准化良好行为企业。重大项目拉动经济发展的作用明显增强。加强优质高端项目招引实施，新批总投资1000万美元以上项目129个，新引进世界500强企业2家，央企项目计划投资额850亿元。创新驱动的能量进一步显现。完善开放式创新体系，推行“政产学研金”合作模式，新建产学研联合体126家、院士工作站8个、省级工程技术研究中心9家、市级以上外资研发机构21家。推动科技成果转化，40个项目列入国家火炬计划，扬子江银杏叶项目获国家科技进步二等奖，春兰高功率镍氢电池研发项目列入国家863计划。抓好知识产权工作，全市专利授权6000件，其中发明专利300件。深入实施“凤城千人计划”，引进各类优秀人才（团队）1230名、海外高层次人才262名，医药高新区3人入选国家第八批“千人计划”。强力推进节能减排，单位地区生产总值能耗降幅全省领先，化学需氧量、氨氮、二氧化硫排放量削减达到序时进度。

城乡建设扎实推进。坚持城乡一体、统筹发展，不断健全规划体系，深入开展“美好城乡建设行动”，城乡面貌、人居环境继续改善。中心城市功能和形象进一步提升。调整完善市区城建体制，加快十大重点工程建设，市区城建投入190亿元。积极实施“畅通工程”，推进城区路网贯通，迎春路东延、凤凰西路西延、育才路等建成通车。市博物馆、图书馆新馆、美术馆、规划展示馆建成开放，市档案馆建成国家二级档案馆，稻河古街区、海军文化公园建设完成序时进度，泰州师专新校区暨高教园区中心共享区、金融服务区、三水湾二期等工程启动建设。完成6个老小区燃气改造，城区水生态环境控制工程竣工，城南第一污水处理厂改扩建建成运营，垃圾焚烧发电厂开工建设。市区公交新辟优化线路9条，建成首末站8个，在高港新建1处公交停车场。加大拆除违法建设力度，推进物业管理进社区，城市管理水平进一步提高。新农村建设深入推进。启动实施“城乡转型2115计划”，重点镇村建设完成投入150亿元。认真办好农村新一轮实事，完成3608个村庄环境综合整治，农村饮水安全工程新增受益人口16.9万人，维修农村低压线路2039公里，新建改造农村公路250公里、桥梁160座，疏浚县乡河道593公里。城乡基础设施建设成效明显。宁启铁路复线电气化改造稳步推进，泰镇高速泰州段获批，阜兴泰高速泰州段立项，引江河二期工程开工建设，卤汀河拓浚、泰东河整治和中小河流治理等工程实施进度加快，电力、邮政、通信等基础设施建设有力推进。生态环境建设力度加大。国家环保模范城市通过复核。新增国家生态乡镇14个、省级生态乡镇21个，姜堰、海陵分别通过省级生态市（区）考核验收和技术评估。强化饮用水源地保护，推进秸秆综合利用，向社会发布PM2.5等6项环境空气质量监测指标。全市新增造林面积9.72万亩，市区新增绿地面积100万平方米。

社会保持文明和谐。制定实施“文化名城建设行动计划”，加强历史文化资源保护，加快文化产业发展，实现文化产业增加值56亿元。文化创意产业综合体项目开工建设，3家企业获批国家文化重点出口企业，新增4个全国重点文物保护单位，望海楼入选中国历史文化名楼，溱潼镇荣获中国民间文化艺术之乡称号。不断完善城乡公共文化服务体系，推进各类公共文化设施免费向市民开放。我市被表彰为全国文化体制改革先进市，兴化被评为省文化先进县，全市8个镇（街）成为省首批公共文化服务体系建设示范镇（街）。成功举办水城水乡国际旅游节、梅兰芳艺术节、全国少儿曲艺大赛等重大文化活动。推进群众性精神文明创建和城乡文明共建，全国城市文明程度指数测评顺利通过，未成年人思想道德建设工作通过省级测评。大力发展教育事业，学前教育五年行动计划深入实施，义务教育优质均衡发展不断加快，高中教育质量稳步提升，靖江市创成省职业教育创新发展实验区，泰州师专专升本工作接受教育部专家组现场考察，3所独立学院获得学士学位授予权，全市基本实现教育现代化。强化医疗卫生服务，基本公共卫生服务范围扩大到10大类41项，对实施基本药物制度的基层医疗卫生机构补助资金1.81亿元；我市创成全国首批基层中医药先进市，靖江、姜堰通过全国农村中医药工作先进单位复核。推进流动人口计生信息管理和均等化服务，在全省率先实现免费孕前优生检查全覆盖。实施“体育惠民工程”，成功举办市第四届运动会，承办中国男子排球联赛、省第七届农运会等重要赛事。加强和创新社会管理。全面实施社会稳定风险评估，深入开展“三解三促”、“四项排查”、党政领导干部接访下访活动，及时妥善解决重点信访问题，我市荣获全省社会管理综合治理先进市，“社会管理创新工程”在全省考评中名列第一。推进平安泰州建设和法治城市创建，加强“六五”普法，高港荣获全国法治创建工作先进单位。全国双拥模范城实现“三连冠”，“三属”抚恤金及义务兵家庭优待金标准实现城乡一体化。突出安全生产监管，深入开展隐患排查治理和“打非治违”专项行动，安全事故起数、死亡人数实现双下降。组织开展食品药品安全专项整治，保障人民群众生命健康安全。海关、国检、海事、边检为地方经济发展作出新贡献，社会科学、广播电视、新闻出版、妇女儿童、民族宗教、外事侨务、统计、气象、防震等工作继续加强，国防动员、民兵预备役和人民防空事业取得新业绩。

人民生活持续改善。城镇居民人均可支配收入27000元，农民人均纯收入12600元，分别增长14%和14.2%。进一步加大就业促进力度，认真实施省完善就业服务体系试点示范项目，新增城镇就业9.1万人、农村劳动力转移3.24万人，城镇登记失业率2.46%。鼓励支持民众创业，新增创业人员5.76万人，带动就业17.32万人，我市被表彰为全国创业先进城市；全市小额担保贴息贷款发放总量突破10亿元，居全省首位。推进社会保障全覆盖，五大保险净增参保13.5万人。全市企业退休职工基本养老金、新农合筹资水平和城乡低保标准进一步提高，职工医保、居民医保最高支付限额分别达30万元和20万元，报销比例平均达80%和70%以上。继续实施全面小康村建设“十百千”提升工程，创成达标村、示范村各100个。启动新一轮扶贫开发，组织实施农村脱贫奔小康工程，新增集体经济年收入20万元以上村400个。认真开展农副产品“保供抑价”工作，对困难群众及时发放物价补贴。出台市区社会救助家庭认定办法、城乡医疗救助实施办法，完善流浪乞讨救助、孤儿基本生活保障自然增长等制度，关心关爱残疾人，加快发展慈善福利事业。着力优化养老服务，推进民办养老机构发展，新建改造居家养老服务站346家，为市区80岁以上老年人发放尊老金。不断完善住房保障体系，全市新建各类保障性住房18930套（间）。住房公积金新增开户4万人、归集金额20亿元。

政务环境不断优化。加大政策引导和扶持服务力度，千方百计稳定经济增长，着力营造良好发展环境。进一步强化政策导向。深入开展调查研究，及时研判经济走势，全面了解掌握行业企业运行动态，适时制定有针对性的对策措施，先后出台了《关于做好当前工作、促进经济持续平稳较快发展的意见》、《关于进一步改善中小微企业发展环境的实施意见》等政策措施。进一步优化政府服务。完善“三服务”活动长效机制，加大要素协调力度，推进服务平台建设，帮助基层和企业排忧解难。实施“中小企业百千万金融服务”工程，开展“小微企业信贷机制建设年”活动，化解企业融资难题。严格控制涉企检查，规范和降低行政事业收费，进一步减轻企业负担。坚持民生优先，全市新增财力的82%用于民生投入，市级财政民生方面的支出占公共财政预算支出的比重达73%。认真办好“12345”政府服务热线、市长信箱、政风行风热线和望海楼实名论坛，政府服务热线提前实现全覆盖，一批事关群众切身利益的热点难点问题得到有效处理。进一步规范施政行为。加快行政审批制度改革，大力推行并联审批，调减行政审批项目278项。实施经济发达镇行政管理体制改革试点，积极推进事业单位分类改革。深化行政权力网上公开透明运行，推进绩效管理，加强效能监察和审计监督。自觉接受人大的工作监督、法律监督和政协的民主监督，主动听取各民主党派、工商联、无党派人士和各人民团体的意见，认真办理人大代表建议和政协提案。严格落实党风廉政建设责任制，着力完善惩治和预防腐败体系，加大监督检查、纠风治乱和案件查处力度。

各位代表，过去一年成绩的取得，是中共泰州市委正确领导的结果，是市人大、市政协关心支持的结果，是全市上下团结拼搏、开拓创新、克难奋进的结果。在此，我代表市人民政府，向全市广大干部群众，向全体人大代表、政协委员，向各民主党派、工商联、无党派人士、各人民团体和广大离退休老同志，向中央和省驻泰机构以及驻泰部队指战员、武警官兵和政法干警，向所有关心支持泰州发展的海内外朋友们，表示衷心的感谢!

我们也清醒地看到，全市经济社会发展中还存在一些矛盾和问题。主要是：经济回升势头尚未根本确立，部分行业、企业经营难度加大，外贸和消费等增长乏力；经济结构性矛盾依然突出，优质高端项目招引和推进难度加大，区域发展存在不平衡性；财政减收增支矛盾突出，城乡居民收入持续较快增长难度加大，民生工作与群众期待相比还有不小差距；政府自身建设还存在薄弱环节，机关作风和行政效能仍需进一步改进，等等。对于这些问题，我们将采取有效措施，认真加以解决。

二、2013年工作任务

各位代表，今年是全面贯彻落实党的十八大精神的开局之年，是推进转型升级综合改革试点、争创全省转型升级示范区的关键一年，也是发展机遇和挑战并存、风险和困难较多的一年。做好今年的政府工作，必须抢抓试点机遇，放大大桥机场和行政区划调整效应，稳中求进、开拓创新、扎实开局，实现经济持续健康发展，保持社会和谐稳定。

今年政府工作的总体要求是：以党的十八大精神为指引，坚持以邓小平理论、“三个代表”重要思想、科学发展观为指导，深入贯彻中央和省经济工作会议、市委四届四次全会决策部署，认真落实省《试点意见》和市《行动纲要》，紧紧围绕主题主线，以提高经济增长质量和效益为中心，以“转型升级突破年”活动为抓手，突出开放创新、综合改革和城乡统筹，认真实施“八项工程”，加快建设“三个名城”，大力发展优势产业、特色园区、重点企业和高端项目，以经济领域、关键环节的率先突破带动城乡、社会全面转型升级，以转型升级、融合发展的新突破为全省“两个率先”作出更大贡献。

全市国民经济和社会发展主要调控目标为：地区生产总值可比增长11%左右；公共财政预算收入增长10%；固定资产投资增长19%；社会消费品零售总额增长15%；实际利用外资14亿美元，外贸进出口总额增长5.5%；城乡居民收入增长12%；城镇登记失业率控制在3.5%以内；居民消费价格总水平不高于全省平均水平；研发经费支出占地区生产总值的比重2.1%；单位地区生产总值能耗下降，化学需氧量、二氧化硫、氨氮、氮氧化物削减完成省定目标任务。

今年将重点做好六个方面的工作：

1. 加快调整产业结构，着力构建富有特色的现代产业体系。把深化产业结构战略性调整作为推进转型升级的主攻方向，进一步发展壮大以“1+3+N”为主导、三次产业协调发展的现代产业体系，不断提升产业发展质态和竞争力。

推进中国医药城跨越发展。坚持以产业化为重点，突出项目核心地位，瞄准国家级科研院所、国际500强企业和国内100强企业，着力招引重大产业化和创新型项目。启动建设中医药产业园，加快阿斯利康、勃林格殷格翰、康淮生物等项目建设，推动杜瑞、亿腾、汇伦等企业早日建成投产，力争项目总量、投资总量和注册总量等重点指标实现翻番，提升高新区在全市经济总量中的比重。加强资本体系建设，推动更多的社会资本与创新型企业嫁接，实现资本与产业的深度融合。放大中国医药城国家产业示范基地和部省共建机制优势，推动全市医药产业和关联产业的整合提升，争取国家、省生物医药产业项目优先在泰州实施，科技成果优先在泰州转化，人才和研发机构优先向泰州集聚，不断增强跨越发展的内生动力。

推进先进制造业高端发展。分解落实“新兴产业双倍增计划”和“传统优势产业转型升级五年规划”年度任务，突出工作重点，细化推进措施。推动高端装备制造、生物技术与新医药、新能源、电子信息等战略性新兴产业和新材料、智能电网、节能环保设备等优势产业集群发展壮大，力争产业规模突破2500亿元。大力发展海洋装备制造、海洋建筑工程、海洋物流仓储等产业，创造条件打造国家级海洋经济创新发展示范区。植入高新技术、加快技术改造、加强品牌建设、鼓励兼并重组，推动传统优势产业高新化发展。支持船舶产业提升主力船型的设计制造能力及配套设备装船率，加快向高技术船舶和特种船舶转型，化工产业向化学新材料转型，金属材料及制品业向金属深加工转型，食品产业重点发展农副产品精深加工和特色食品。推进工业化与信息化“两化”融合，深化示范企业和示范区创建，鼓励重点企业率先推广应用物联网技术。进一步提高建筑业发展水平，推动建筑企业管理创新、资质升级，建筑业总产值增长12%。

推进现代服务业提速发展。围绕加快建立现代服务业区域中心，重点打造“四大板块”，促进现代服务业集聚区提档升级，新增百亿级集聚区1-2家，建成省级现代服务业示范区1家。发挥沿江区位优势，按照“港口、仓储、加工、交易”四位一体的发展思路，大力构建国际制造业基地和区域性现代物流基地，推动先进制造业和现代服务业深度融合。加快核心港区功能化建设，启动实施“泰州公路港”物流项目。积极发展信息服务、现代物流、工业设计、金融及融资担保、科技与咨询服务等生产性服务业，推动企业从制造环节向服务环节拓展。放大“水城水乡”和特色文化资源优势，加快建设休闲型文化生态旅游区，大力发展乡村和工业旅游。

推进现代农业高效发展。深入实施“5218”提升工程，加快建设国家现代农业示范区。加强规划统筹，整合海陵、姜堰、泰州农业开发区优质农业资源，合力打造中心城市北部现代农业走廊。推进现代农业产业园区、农产品加工集聚区、农产品市场体系和质量安全体系建设，新增省级园区2-3家、省级以上龙头企业6家、“三品一标”50个以上。突出水稻机插和秸秆还田，组织开展农业机械化推进年活动，农业综合机械化水平达82%。实施现代农民培育、科技进村入户和农业“三新”工程，科技对农业贡献率提高到65%左右。加强高标准农田、高效节水灌溉工程等农田水利基础设施建设，提高农业综合生产能力。坚持“守住一条底线”、“抓住两个关键”，积极创新农业生产经营体制机制，着力构建集约化、专业化、组织化、社会化相结合的新型农业经营体系。

2. 强化开放创新驱动，创建新的竞争优势。把“双轮驱动”作为推进转型升级的强大引擎，坚持在开放中高起点创新、在创新中高起点开放，努力在更广范围、更宽领域、更高层次上集聚高端项目、优势产业和创新要素。

全面提高开放型经济水平。进一步加大招商力度，加强与知名央企和跨国公司的紧密协作，积极招引投资体量大、科技含量高、支撑效应强的高端项目。坚持综合推介与专题活动相结合，继续办好医博会、科洽会等重大活动，既注重提高大活动招商成效，更注重增加小分队招商频次，全年力争引进超3000万美元项目20个，其中过亿美元项目3个。支持企业稳定传统市场、开拓新兴市场，加大对外贸重点行业、重点企业、重点地区的扶持力度，大力吸引大型外贸企业和外商代表处落户泰州。加快企业“走出去”步伐，鼓励开展境外投资、发展国际贸易、承包境外工程。加强外事、侨务和港澳台事务工作，扩大对外交流合作。

精心打造高品质特色产业园区。推动各类产业园区扩容提质，注重加强品牌建设和特殊功能区、公共服务平台建设，重点培育20个在国内外有影响、集聚效应明显、产业特色鲜明的专业园区。推动靖江经济开发区发挥国家级开发区优势，突出项目建设、功能提升和特色打造；推动市经济开发区突出优势主导产业培育，加快向以光电产业为主导的国家级出口加工区和综合保税园区转型；推动新能源产业园区针对新能源产业发展的新特点，加快项目引进集聚，把企业培大育强。推进沿江开发和跨江联动开发，提升江阴—靖江工业园区产业发展层次和规模。

认真组织实施转型升级重大项目“双百工程”。改进项目招引和推进方式，提高环保准入门槛，实行专业评估，促进项目发展高端化。推进中海油气、东联化工、纬创二期、智航锂离子材料、诚达精工、方泰超纤、甲醇制烯烃一体化等项目加快建设。整合各类产业项目扶持资金，加大对新兴产业和技术改造项目的补助、奖励力度，引导各类上争资金向“双百工程”倾斜，确保新兴产业、先进制造业和现代服务业投资增速高于固定资产投资增速。

加快构建区域创新体系。深化与中科院等高校院所的战略合作，加快建设全省重要的产学研合作创新集聚区。新建产学研联合体100家，实施高质量合作项目350个，高新技术产业产值占规模以上工业比重提高到36%。大力培育创新型企业，支持企业建设工程研究中心、技术中心，推进规模以上工业企业拥有专利、大中型工业企业自建研发机构两个“全覆盖”。支持企业主持或参与制修订国家和行业标准，加强商标注册和保护，争创驰名、著名商标。加快完善各类科技创新服务平台，放大国家专利战略推进与服务（泰州）中心效应，鼓励引导行业骨干企业建立产业技术创新联盟。大力实施“科技金融创新计划”，设立科技金融贷款风险补偿金，扩大科技小额贷款公司试点范围，推进知识产权质押、科技保险、科技担保和股权融资。

3. 充分发挥企业主体作用，不断发展壮大实体经济。把企业发展作为推进转型升级的坚实基础，创新有利于实体经济发展的政策措施，推动生产要素、政策资源、政府服务更多地向企业集中。

推进重点企业发展。密切关注大中型企业尤其是“50强”企业运行状况和发展走势，为企业融资、市场开拓、项目建设、人才招引创造有利条件。深入实施“千企升级百企示范工程”和“大企业（集团）培育计划”，大力扶持具有发展潜质的骨干企业和成长型企业，加快培育具有国际竞争优势、处于国内行业领先位置的本土企业集团，力争全市年销售超50亿元的企业达20家。积极推进“百企上市培育工程”和企业直接融资，新增上市企业1-2家。

扶持中小微企业发展。组织实施高成长中小企业培育和小微企业上规模培育工程，努力提高整体素质和竞争力。推动中小企业向“专精特新”方向发展，加大对初创期、成长期科技型中小企业融资支持和财政政策扶持力度。落实调低小微企业所得税税率等政策措施，降低强制性产品认证办理费用，切实减轻企业负担。推行“富民阳光信贷”，加大金融服务实体经济力度，确保中小微企业信贷投放增速不低于全部贷款的平均增速。放手发展民营经济，鼓励引导民间资本进入基础产业、基础设施、市政公用事业和政策性住房建设等领域。

加强人才队伍建设。围绕建成全省“双创计划”重要人才基地目标，深入实施“12368”人才工作纲要和“凤城千人计划”，举办好“海外博士泰州行”等活动，探索构建开放式、柔性化引才机制，确保全年引进培养高层次创新创业人才（团队）1300名、海外优秀人才280名。强化人才特区体系建设，推动医药城创建“省级人才特区”、各市（区）建立特色产业园“人才特区”。面向社会需求，加强职业技术教育，打造区域性高技能人才培养基地。

4. 更大力度推进改革，增强发展动力和活力。抓住机遇、先行先试，更加自觉、更加坚定地推进改革，大力破除各类要素制约和体制机制障碍，以改革的率先突破保障转型升级的新突破。

推进行政管理体制改革，加快转变政府职能。推进转型升级综合改革试点，行政管理部门首先要“自我革命”。深化行政审批制度改革，市场竞争机制能够有效调节的，公民、法人或其他组织能够自主决定的，行业组织或中介机构能够自律管理的事项，一律不设定行政审批；可以采用事后监管和间接管理方式的事项，一律不再搞前置审批。对现有审批事项进行全面清理，大力推进并联审批、网上审批，确保“三集中、三到位”落到实处，最大限度地减少审批事项、降低审批收费、压缩审批时限、提高审批效率。把产业项目和为民服务事项的审批改革作为重中之重，加快“绿色通道”建设。加强公共资源交易平台建设，进一步规范交易行为。理顺市区行政管理和建设管理体制，深化经济发达镇行政管理体制改革，推行对重点园区和重点镇的简政放权改革试点，推进政府行业管理、社会生活事务管理等职能向社会组织转移，扩大政府购买公共服务范围。

推进重点领域和关键环节的体制机制改革，加快转变经济发展方式。围绕资源要素市场化改革，鼓励金融创新，开展民间融资改革试验，探索建立民间资本管理服务的新机制；以建立土地规范有序流转制度为重点，完善土地资源开发利用机制，探索建立土地承包经营权流转市场。围绕科技创新体制改革，加快建立项目立项机制、人才激励机制、紧密型产学研合作机制和政府对科技投入的评价支持机制。围绕绿色发展机制改革，建立完善体现生态文明要求的目标体系、考核办法、奖惩机制，并在资源环境价格改革、排污权有偿交易试点、绿色信贷等方面重点突破。围绕区域统筹发展机制改革，推行“飞地”发展模式，在管理上以市（区）为单位加强统筹，建立超越行政区域的协调机制以及科学可行的政绩考核、财力分成和工作推进机制。加强财政预算绩效管理和社会综合治税，探索建立财政资金“拨改贷”、“拨改投”新模式，做好“营改增”和结构性减税相关工作。

5. 扎实推进城镇化，推动城乡发展一体化。把城镇化和“三个名城”建设作为推进转型升级的重要内容，着力推进城乡规划、基础设施、公共服务等方面的一体化，加快构建特色鲜明、功能互补、和谐融合的现代城乡形态。

提升中心城市形象品位。进一步优化城市布局。启动修编城市总体规划，推进各城市片区基础设施建设的有机统一和重大公共服务设施的共建共享，推进海陵、高港、姜堰和高新区之间的交通互联、功能互融，形成科学合理的城市空间资源和生产力布局。进一步完善城市功能。组织开展城市综合交通整治，加强公共停车场建设，实施汽车西站迁建，改造“九路一桥”，建设“五纵五横”景观道路，推进骨干道路贯通，加快形成城市外环快速交通体系。加大凤城河风景区、金融服务区等重点项目建设力度，加快建设滨江新城。推进老小区和农贸市场改造建设，扩大市区污水管网覆盖范围。继续发展公交事业，力争市区公交出行分担率提高3个百分点。加强高新区六大功能区建设，实施高教园区中心共享区、新区高级中学、人民医院新院、体育公园等重点工程，加快建设产城一体的医药名城。进一步推动城市精致化发展。加快城市重要节点的改造建设，实施重点路段立面出新和环境整治。加强城市管理，着力提高数字化、网格化、精细化水平。

强化重大基础设施建设。加快宁启铁路复线电气化改造，开工建设泰镇高速泰州段，争取京沪高速泰州段扩容和阜兴泰高速泰州段及早实施，启动建设328国道九龙段、333省道兴化段改扩建和231省道泰州至高港段改线，抓紧实施334省道泰兴段。改造广靖高速靖江互通，积极开展泰常过江通道、盐泰锡宜城际铁路等重点项目前期工作。推进泰州电厂二期、国家特高压输变电工程等项目建设。完成卤汀河拓竣，推进引江河二期、泰东河整治等重点水利工程建设，启动周山河整治三期工程。

推进城乡统筹协调发展。扎实开展“美好城乡建设行动”，深入实施“城乡转型2115计划”，加快建设转型发展示范小城市、示范村和新型农村社区，鼓励支持市区和靖江率先探索，加快成为推进城乡一体化的先行区。有序引导农民居住向新型社区或城镇集中、工业企业向园区集中、农业用地向规模经营集中，统筹推进公共交通、区域供水、燃气供应、污水垃圾处理、消防站点等基础设施建设。深入开展村庄环境综合整治，年内完成2500个村庄的整治任务。进一步深化户籍制度改革，全面推行居住证制度，放宽农民进城和外来人口落户限制。深入实施全面小康村建设“十百千”提升工程和脱贫奔小康工程，加大对黄桥老区和里下河地区资金、政策支持力度。

加强生态环境建设。深入实施“生态文明建设工程行动计划”，开展系列创建活动，加快生态名城建设。靖江、泰兴建成国家环保模范城市，兴化通过省级考核；海陵、姜堰生态市（区）创建工作力争通过国家验收，靖江、高港建成省级生态市（区），泰兴通过省级技术评估，全市基本达到省级生态市考核标准。实施“清水、蓝天、净地”工程，加强水域、农田、湿地、绿地等生态系统保护，建设中心城市北部生态区、原生态长江绿带、大桥北接线两侧林带和里下河湿地风光带。严格控制水资源管理“三条红线”，创成国家级节水型社会建设示范市。突出抓好节能减排，推进清洁生产和环境管理体系认证，严格落实耕地保护责任制和土地节约集约利用制度。

6. 加强社会建设和管理，增进民生幸福、社会和谐。把保障改善民生、构建和谐社会作为推进转型升级的价值取向，把人民利益放在第一位，按照“守住底线、突出重点、完善制度、引导舆论”的思路，加快公共服务体系建设，努力让人民群众过上更加美好的生活。

千方百计增加居民收入。深入实施“城乡居民收入六年倍增计划”，着力促进农民增收，进一步拓宽就业惠民、创业富民、投资兴民、社保安民、帮扶助民“五大增收渠道”。围绕重保障、促平等、强素质，实现更高质量的就业，注重抓好高校毕业生、城镇就业困难人员和农村转移劳动力就业工作。大力推进全民创业，实施能力提升工程，开展助推“1+1”活动。全年净增城镇就业4.6万人，新增自主创业5万人、创业带动就业15万人。鼓励引导市民投资兴业、置办物业，提高群众的资产性收入。统筹城乡社会保障体系建设，整合城乡居民养老和医疗保险制度，加强被征地农民社会保障，推进农民工工伤保险全覆盖，发展多层次的养老服务，完善残疾人保障服务体系，支持发展慈善事业，建成社会保险、社会福利、社会救助相衔接的保障网。加强大病救助，防止因病致贫、因病返贫。健全住房保障体系，全市新开工保障性住房7500套（间）。扩大住房公积金制度覆盖面，新增缴存职工3万人。

全面推进社会进步。统筹发展文化事业与文化产业、城市文化与农村文化、精品创作与群众文化，加快文化名城建设。加强各类历史文化资源的保护和合理利用，做好中国历史文化名街申报工作。推进稻河古街区和口岸水景街区、天德湖中医药文化主题园、评书评话和盐税文化主题博物馆建设，加快文化创意产业园、海军文化公园、凤城河文商创意园建设。加大文化惠民力度，完善文化服务体系，加快镇（街）文化中心、村（居）特色文化广场建设，进一步扩大公共文化设施免费开放范围。加强社会主义核心价值观教育，深化全国文明城市创建，提高市民素质和社会文明程度。精心办好水城水乡国际旅游节、梅兰芳艺术节等重要节庆活动和“泰州学派”学术研讨会等文化学术活动。组织实施“163”行动计划，深化不同学段教育改革发展。加大学前教育投入力度，提高优质幼儿园比例；推进义务教育优质均衡发展，海陵、姜堰力争创成省级示范区；进一步提高高中教育质量，加强职业教育国际合作，加快发展高等教育。深化医药卫生体制改革，巩固完善基本药物制度，稳步实施公立医院改革试点和市区医疗卫生资源整合，推进专科医院和基层医疗卫生机构建设，进一步完善医疗体系、规范诊疗行为、提升服务水平。广泛开展全民健身、青少年阳光体育运动，备战省第18届运动会。推进出生人口性别比综合治理，扎实开展人口协调发展先进市（区）创建。加强统计“四大工程”建设，组织好第三次全国经济普查。加快气象现代化建设，建成气象综合探测基地。认真做好史志档案、社会科学、新闻出版、妇女儿童、民族宗教、人民防空、防震等工作。

加强和创新社会管理。进一步强化社会管理体系建设，加快完善县、乡、村三级管理服务平台，夯实政法综治基层基础工作，不断提升社会管理科学化水平。完善社会稳定风险评估、领导干部接访下访工作制度，深化“三解三促”和矛盾纠纷排查化解，从源头上解决突出信访问题。整合“12345”政府服务热线和政风行风热线，加强热线与市长信箱、望海楼实名论坛的互动融合，进一步畅通群众诉求表达、建言献策渠道，加快建立规范统一的受理、跟踪、反馈和督查考核机制，着力推动民生热点难点问题的解决。强化社会治安综合治理，完善立体化防控体系，严厉打击各类违法犯罪活动，提高人民群众安全感。深化法治城市创建，加强普法宣传教育和法律援助。推进农村基层民主法制建设，组织好第十届村（居）委会换届选举。落实安全生产措施，强化隐患排查治理，严防重大安全事故。实施“放心工程”，加大监管力度，保证群众饮食用药安全。深化双拥共建，做好国防教育、国防动员、民兵预备役和兵员征集工作。

各位代表，推进转型升级综合改革试点、争创全省转型升级示范区，对政府工作提出了新的更高要求。我们将扎实推进学习型、服务型、创新型政府建设，以能力素质、行政效能的大提升推动转型升级的新突破。始终坚持科学施政。紧紧围绕省《试点意见》和市《行动纲要》，加强对重大任务、重要事项、重点课题的调查研究，进一步理清思路、明晰路径、创新举措。健全行政决策机制，把政治协商纳入决策程序，增强民主协商实效性。强化政府组成部门工作责任，对转型升级重点工作实行目标化管理、项目化推进和优质化服务。始终坚持依法行政。自觉接受人大的法律监督、工作监督和政协的民主监督，重视新闻舆论和社会公众监督，加大审计监督力度。深化和扩大政务公开，完善政府信息发布制度，提升行政权力网上公开透明运行水平。加强绩效管理、效能监察和行政问责，提高政府执行力和公信力。始终坚持廉洁从政。严格执行中央关于改进工作作风、密切联系群众的八项规定和省十项规定，大力弘扬求真务实作风，出实招、办实事、求实效。牢固树立艰苦奋斗、勤俭节约思想，严格控制一般性支出，把有限财力更多地用在保障和改善民生上。坚持标本兼治、综合治理、惩防并举、注重预防方针，全面推进完善惩治和预防腐败体系建设，更加科学有效地防治腐败，严格执行党风廉政建设责任制，严厉查处各类违法违纪行为，树立为民务实清廉的良好形象。

各位代表，泰州的发展迎来了难得的历史机遇，具备了更好的基础和优势，站在了更高的起点上。让我们紧密团结在以习近平同志为总书记的党中央周围，以党的十八大精神为指引，高举中国特色社会主义伟大旗帜，在省委、省政府和市委的坚强领导下，同心同德、攻坚克难、扎实工作，不断开创科学发展新局面，为把泰州建设得更加美好而努力奋斗
